# Minimally Invasive Total Versus Partial Thymectomy for Early-Stage Thymoma

**DOI:** 10.3390/cancers17152518

**Published:** 2025-07-30

**Authors:** Alexander Pohlman, Bilal Odeh, Irene Helenowski, Julia M. Coughlin, Wissam Raad, James Lubawski, Zaid M. Abdelsattar

**Affiliations:** 1 Stritch School of Medicine, Loyola University Chicago, Maywood, IL 60153, USAihelenowski@luc.edu (I.H.); julia.coughlin@lumc.edu (J.M.C.);; 2 Department of Thoracic and Cardiovascular Surgery, Loyola University Medical Center, Maywood, IL 60153, USA; 3 Department of Surgery, University of Illinois Chicago, Chicago, IL 60612, USA; 4 Department of Veterans Affairs, Edward Hines Jr. VA Hospital, Hines, IL 60141, USA

**Keywords:** thymoma, total thymectomy, partial thymectomy, minimally invasive surgery

## Abstract

**Background/Objectives**: Total thymectomy is currently the gold standard operation for treating thymoma. However, recent studies have suggested the potential health consequences of thymus removal in adults, including possible increased autoimmune disease and all-cause mortality. In this context, we assess oncologic outcomes following total vs. partial thymectomy for early-stage thymoma. **Methods**: We identified patients diagnosed with WHO types A–B3 thymoma between 2010–2021 from a national hospital-based dataset. We excluded patients with stage II or higher disease, open resections and perioperative chemo-/radiation therapy. We stratified patients into total and partial thymectomy cohorts. We used propensity score matching to minimize confounding, Kaplan–Meier analysis to estimate survival, and Cox proportional hazards to identify associations. **Results**: Of 1598 patients with early-stage thymoma, 495 (31.0%) underwent partial and 1103 (69.0%) total thymectomy. Patients undergoing partial thymectomy were similar in sex (female 53.7% vs. 53.4%; *p* = 0.914), race (white 74.5% vs. 74.0%; *p* = 0.921), comorbidities (0 in 77.0% vs. 75.5%; *p* = 0.742), and tumor size (48.7 mm vs. 50.4 mm; *p* = 0.455) compared to total thymectomy. There were no differences in 30-day (0.8% vs. 0.6%, *p* = 0.747) or 90-day mortality (0.8% vs. 0.8%, *p* > 0.999), which persisted after matching. Moreover, 10-year survival was similar in both unmatched (*p* = 0.471) and matched cohorts (*p* = 0.828). Partial thymectomy was not independently associated with survival (aHR = 1.00, *p* = 0.976). **Conclusions**: In patients with early-stage thymoma, partial and total thymectomy were associated with similar short- and long-term outcomes. In light of recent attention to the role of the thymus gland, the results add important insights to shared decision-making discussions.

## Simple Summary

Total thymectomy is the current standard for the treatment of early-stage thymoma. However, recent studies have raised partial thymectomy as an option and brought attention to the thymus’s potential role in protection against autoimmune disease, development of extra-thymic malignancy and overall mortality. Therefore, it’s unclear if an organ-sparing approach is an equivalent alternative. In this context, we compare partial versus total thymectomy for early-stage thymoma using a national database. We demonstrate that partial- and total-thymectomy have similar short- and long-term outcomes. These results may help better inform patients of their surgical options within shared decision making discussions with their surgeon.

## 1. Introduction

The role of the thymus gland in adults is not clear, particularly since the thymus naturally involutes with age. However, a recent study in the *New England Journal of Medicine* (*NEJM*) examined the health consequences of thymectomy, demonstrating worse all-cause mortality and higher rates of autoimmune disease [1]. However, ability to definitively draw these conclusions is limited by the design of the study. In patients with thymoma, the Society of Thoracic Surgeons currently recommends total thymectomy for all thymomas, except in high-risk patients in whom a partial thymectomy may be considered [2]. The rationale behind total thymectomy stems from previous studies that demonstrated that all fatty tissue may harbor microscopic foci of thymic cells, which could give rise to recurrent thymoma [3].

With the newfound interest in preserving the thymus, understanding the comparative effectiveness of a partial thymectomy (also referred to as a thymomectomy) compared to a total thymectomy (also called a thymothymectomy) is needed on two fronts. The first of these concerns the question of whether total thymectomy is truly a better oncologic operation for early-stage thymoma compared to partial thymectomy. Previous studies on this topic have shown mixed results. For example, a multicenter, international study revealed similar post-op complication rates and slightly improved five-year survival and freedom from recurrence with total thymectomy [4]. However, in that study, only 6% of patients underwent partial thymectomy. Studies with larger sample sizes showed similar five-year disease-free survival [5,6,7,8]. However, these studies included heterogeneous populations, various foreign institutions, and a blend of surgical techniques (minimally invasive and open), and they spanned multiple decades due to the rarity of the disease. Therefore, these results are not easily generalizable, which, in conjunction with the mixed nature of the results, led to poor evidence for current practice. On the other hand, the recent findings of increased all-cause mortality associated with total thymectomy may be over-emphasized, as discussed in a recent review of the literature [9]. This leaves surgeons without clear evidence-based recommendations for current practice; they are left to decide between two opposing theoretical benefits: remove the entire thymus to prevent recurrence or preserve part of the thymus to prevent the reported occurrences of extra-thymic malignancy and/or auto-immune disease.

In this context, we conduct a comparative effectiveness study using the largest hospital-based national dataset on this topic. Specifically, we examine short- and long-term outcomes in patients with early-stage thymoma undergoing a minimally invasive partial or total thymectomy. We hypothesize that partial thymectomy will have similar long-term outcomes to total thymectomy. The results are important for shared decision making for patients afflicted with thymoma.

## 2. Materials and Methods

### 2.1. Data Source

This study was reviewed and deemed exempt by the Loyola University Chicago institutional review board (IRB 219066, 10 January 2025). For this retrospective cohort study, we used data from the National Cancer Database (NCDB), which is a prospective national cancer registry that collects data from ~1500 Commission on Cancer accredited centers across the US, capturing ~70% of all cancer cases. The thymus malignancy participant user file was utilized and included patient demographics, tumor characteristics (such as histology and size), tumor node metastasis (TNM) staging, administered treatment, and short- and long-term outcomes associated with each case [10].

### 2.2. Patient Population

**Figure 1:**
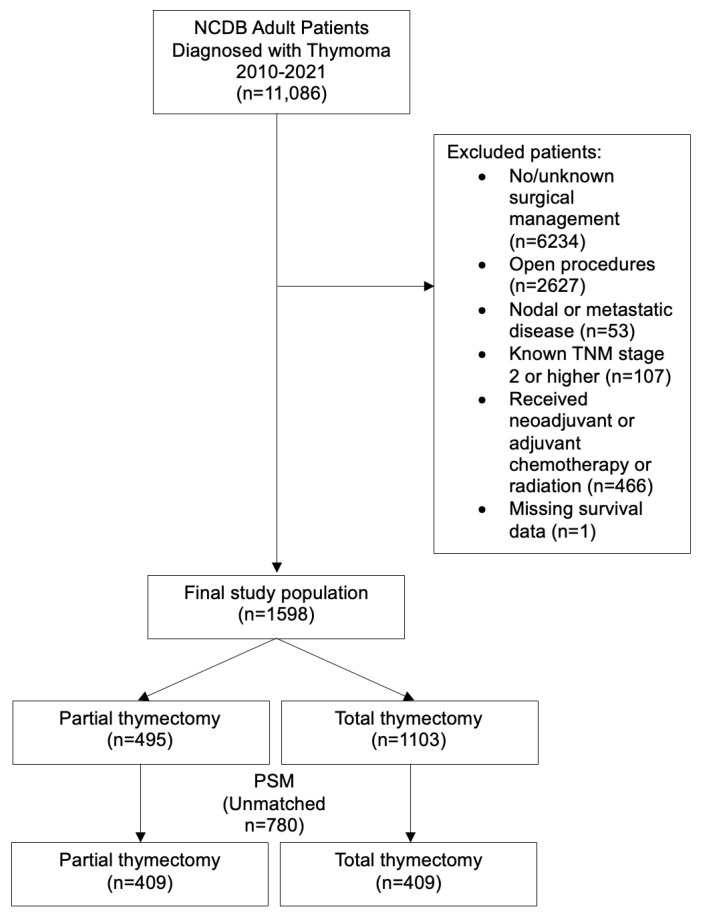
Consort diagram for the creation of the study population. NCDB—National Cancer Database, TNM–tumor node metastasis.

We identified adult patients with World Health Organization (WHO) types A–B3 thymoma using site code C379 and histology codes 8580–8585 undergoing partial or total thymectomy (site-specific surgery codes 30 and 40, respectively). All patients underwent minimally invasive surgery. Any patients with invasive disease requiring en bloc resections were not included. Patients with TNM stage II or higher were excluded. The NCDB does not contain Masaoka staging data. In patients without complete American Joint Committee on Cancer (AJCC) staging data and to ensure the inclusion of only stage I disease, any patients with nodal or metastatic disease and perioperative chemo- or radiation-therapy were excluded. Open resections were also excluded, as these are more likely to be associated with total thymectomy and thus may confound results. Figure 1 shows a consort-type algorithm.

### 2.3. Main Exposure and Independent Variables

The main exposure variable was the extent of resection. Patients were stratified into two cohorts: partial thymectomy and total thymectomy. Other independent variables included age, sex, race, ethnicity, comorbidity index, tumor size, histology, primary insurance payer, median income quartiles, and facility type (academic vs. nonacademic).

### 2.4. Outcome Variables

The primary long-term outcome of this study was 10-year overall survival, measured in months from initial diagnosis. Short-term outcomes were 30-day mortality, 90-day mortality, resection margins, length of stay, and unplanned readmission within 30 days. Resection margins were categorized as either negative (R0) or positive (R1/R2). Resection margins were drawn from final pathology reports for each case.

### 2.5. Statistical Analysis

Baseline clinical and demographic characteristics were compared between partial and total thymectomy groups using Fisher’s exact tests for categorical variables and the Wilcoxon rank–sum test for continuous variables.

Short-term outcomes, including 30-day mortality, 90-day mortality, resection margins, length of stay and unplanned readmission, were compared between the two groups. The Wilcoxon rank–sum test was used to assess differences in length of stay and Fisher’s exact tests, or Chi-squared tests in computationally intensive cases, were used for differences in mortality, resection margins, and readmission.

Individual factors affecting long-term survival were compared using Cox proportional hazards analysis on unmatched data. Factors affecting survival in the Cox analysis, in addition to basic tumor and patient characteristics, were included in the propensity score match; this included age, sex, race, ethnicity, comorbidities, insurance status, median income, histology, tumor size, and facility type. These were used in a multivariable probit regression to calculate propensity scores for each patient. Patients in both groups were matched 1:1 using the nearest neighbor method without replacement. A caliper of 0.5 was used, as this was just below 20% of the propensity score standard deviation, which is consistent with prior studies and recommendations [11,12]. All standardized bias was reduced to |<10%|, indicating balance. Unmatched patients were dropped from the analysis.

After matching, short-term outcomes were again compared with the same statistical tests used on the unmatched groups. Long-term outcomes were compared using Kaplan–Meier survival analysis in both unmatched and matched groups to estimate 5- and 10-year survival with a log-rank test for significance.

Statistical analyses were performed using Stata version 17.0SE (StataCorp LLC, College Station, TX, USA) and R 4.3.1 (R Foundation for Statistical Computing, Vienna, Austria). All tests were two-sided using a *p* value < 0.05. Confidence intervals were reported to a 95% confidence level.

## 3. Results

After fitting the inclusion criteria, there were 1598 patients with early-stage thymoma who underwent minimally invasive surgery. Of those, 495 (31.0%) underwent partial thymectomy and 1103 (69.0%) underwent total thymectomy. The robotic approach was more common (69.8% of cases) than thoracoscopy. Patients undergoing partial thymectomy in the unmatched cohorts were similar in terms of sex (female 53.7% vs. 53.4%; *p* = 0.914), race (white 74.5% vs. 74.0%; *p* = 0.921), comorbidities (0 in 77.0% vs. 75.5%; *p* = 0.742), and tumor size (48.7 mm vs. 50.4 mm; *p* = 0.455) compared to total thymectomy. Age was slightly higher in the partial thymectomy group (63.7 vs. 61.1 years old, *p* < 0.001). Academic centers tended to have a higher proportion of partial thymectomies than nonacademic centers (33.2% vs. 28.8%), but this was not statistically significant (*p* = 0.058). A full summary of the demographic and clinical characteristics of the two groups can be seen in Table 1.

### 3.1. Unmatched Analysis

All short-term outcomes were similar between partial and total thymectomy groups. There were no differences in 30-day mortality (0.8% vs. 0.6%, *p* = 0.747), 90-day mortality (0.8% vs. 0.8%, *p* > 0.999), length of stay (2 days vs. 2 days, *p* = 0.943), unplanned readmissions (1.6% vs. 2.2%, *p* = 0.564), or positive margin rates (9.9% vs. 8.5%, *p* = 0.386). All short-term outcomes can be seen in Table 2.

Next, Cox proportional hazards analysis was used to see how individual variables affected overall survival, as shown by the adjusted hazard ratios (aHR) listed in Table 3. Partial thymectomy had no impact on survival compared to total thymectomy (aHR 1.00, *p* = 0.976). Factors associated with worse survival included increasing age (aHR = 1.06, *p* < 0.001), a Charlson–Deyo comorbidity index of 2 + (aHR 2.91, *p* < 0.001) and Hispanic ethnicity (aHR 2.20, *p* = 0.022). On the other hand, undergoing thymectomy at an academic center was associated with improved survival (aHR 0.66, *p* = 0.030). Kaplan–Meier survival analysis in the unmatched cohorts revealed no difference in 10-year survival between the two groups (*p* = 0.471).

When specifically comparing robotic to thoracoscopic thymectomies, there were only minor differences in demographics and tumor characteristics. The two groups were similar in terms of age (62.0 vs. 61.9, *p* = 0.962), sex (female 54.0% vs. 52.3%, *p* = 0.548), race (white 74.5% vs. 73.4%, *p* = 0.862), comorbidities (Charlson–Deyo Index = 0 in 74.6% vs. 79.0%, *p* = 0.131), and tumor size (48.9 vs. 53.0 mm, *p* = 0.209). The only statistically significant difference was in the primary insurance provider (private: 48.4% robotic vs. 43.8% thoracoscopic, *p* = 0.032). There were higher readmission rates in the thoracoscopic group (3.1% vs. 1.5%, *p* = 0.035), but all other short-term outcomes were similar, including 30-day mortality (0.8% vs. 0.4%, *p* = 0.521), R0 resections (91.8% vs. 89.3%, *p* = 0.125), and median length of stay (2 vs. 2 days, *p* = 0.978). All demographics and short-term outcomes of the robotic and thoracoscopic groups can be seen in Supplemental Table S1.

### 3.2. Propensity Score Matched Analysis

After propensity score matching, the two groups were similar in all measured areas, and all measured standardized bias was <|10%|. The Love plot comparing standardized bias before and after matching is shown in Supplemental Figure S1, and bihistograms of propensity scores before and after matching are shown in Supplemental Figure S2. All demographics and the standardized bias of the matched cohorts can be seen in Table 1 for comparisons to unmatched groups.

The similarities in short- and long-term outcomes persisted after matching. Perioperative outcomes, including 30- and 90-day mortality were similar (1.0% vs. 1.0%, *p* > 0.999) as were length of stay (2 days vs. 2 days, *p* = 0.315) and unplanned readmission rates (1.7% vs. 1.7%, *p* > 0.999). Full results can be seen in Table 2 for comparison to unmatched data.

**Figure 2:**
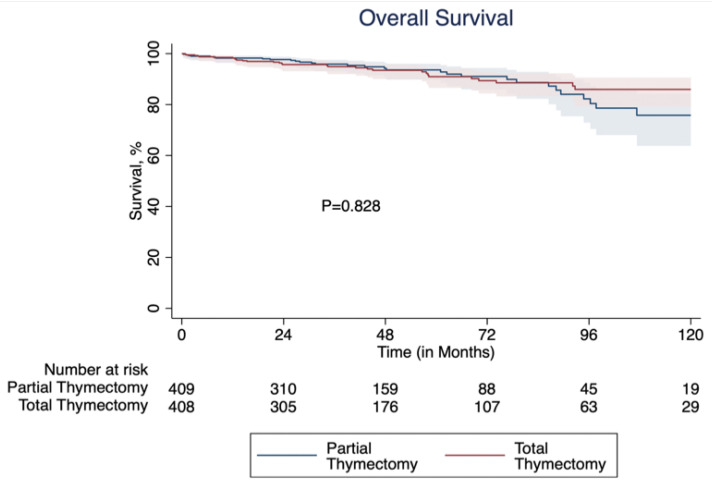
Kaplan–Meier survival curve comparing 10-year survival of partial versus total thymectomy after propensity score matching.

Oncologic outcomes were similar in the matched cohorts. R0 resection rates were similar (90.3% vs. 89.9%, *p* = 0.905), as were 5- and 10-year survival rates, as shown in Figure 2. Survival at 5 years was 93.6% partial vs. 90.8% total thymectomy (*p* = 0.237) and at 10 years it was 75.8% partial vs. 85.8% total (*p* = 0.828).

## 4. Discussion

In this national hospital-based study of patients with early-stage thymoma undergoing minimally invasive surgery, we found that partial thymectomy had similar peri-operative outcomes, similar rates of R0 resection, and similar 5- and 10-year survival when compared to total thymectomy. Thus, partial thymectomy can be viewed as a safe and effective operation for early-stage thymoma. Likewise, patients undergoing total thymectomy did not have worse overall survival as a consequence of the surgical removal of the thymus, as suggested by recent studies [1]. These findings are important for shared decision making, particularly at a time where the role of the thymus is being revisited.

In this study, short-term outcomes were nearly identical between partial and total thymectomy. In undertaking this study, we did not think there would be a difference in perioperative outcomes, as all included patients underwent a minimally invasive operation for early-stage disease by design. The low peri-operative mortality rate of ~1% and short length of stay are similar to those reported in other studies that have established the role of minimally invasive thymectomy [13]. In addition, rates of positive margins did not differ between groups and are similar to those found in previous studies [14]. Complete resection and negative margins have previously been associated with improved long-term survival and decreased recurrence [15,16]. It is important to note that previous studies have shown no difference in rates of negative margins between open and minimally invasive techniques, so we would not expect the inclusion of open cases to change these results [14,17].

The primary outcome in this study was long-term survival. In the present study, 5- and 10-year overall survival were similar in patients undergoing partial versus total thymectomy, and there was no difference in the multivariable Cox proportional hazards analysis (partial thymectomy aHR = 1.00). It is important to note that 10-year survival was slightly lower in the partial thymectomy group (75.8% vs. 85.8%), but the difference was not statistically significant, and the reverse was true at 5 years (93.6% partial vs. 90.8% total thymectomy). As this is overall survival and not cancer-specific or disease-free survival, it is difficult to comment further on the cause of this finding. However, given that the two survival curves cross at several time points, significance is not likely. Prior studies are in line with our findings of similar survival, although they were limited to non-homogenous populations, a mix of open and minimally invasive techniques, and a smaller proportion of partial thymectomies [8,18,19]. Thus, our study adds more evidence to existing literature, particularly regarding minimally invasive thymectomy.

The Society of Thoracic Surgeons’ consensus statement states that patients who are high-risk for surgery may undergo a partial thymectomy rather than a total thymectomy [2]. This recommendation may stem from other studies that have shown fewer post-operative complications, such as decreased pleural drainage and decreased hospital stay with partial thymectomy [18]. While we are not able to capture these specific complications in our study, we found that hospital length of stay and perioperative mortality were identical. Again, this is not surprising, as both groups underwent a minimally invasive operation. In addition, both groups had similar comorbidity burdens prior to surgery.

There may be other benefits outside of the surgical and oncologic treatment course for preserving part of the thymus. A recent study in the NEJM found that removal of the thymus gland was associated with increased rates of autoimmune disease, extra-thymic malignancy and all-cause mortality [1]. However, there is concern that the results of this NEJM study may be over-interpreted.

A review by Kaminski et al. highlights important methodological issues within the NEJM study, including selection bias and potential confounding factors [1,9]. The NEJM article was a case–control study that compared patients undergoing cardiac procedures to those undergoing thymectomy, as is typically for thymic malignancy. Therefore, this is a non-comparable control group that does not help us to identify how the thymus or associated thymic pathology directly affects mortality, extra-thymic malignancy or autoimmune disease. Additionally, most deaths occurred within one year of surgery, indicating that a mortality difference was likely a product of the innate risks of surgery rather than a protective effect of the thymus. Finally, a high percentage of patients developed autoimmunity, likely indicating pre-existing bias in the study population [9]. Given these concerns, the role of the thymus in adults is still unclear. However, regardless of the ongoing debate surrounding the role of the thymus, if an organ-sparing approach does not compromise oncologic outcomes, then it would make sense to consider organ preservation as an option during discussion with patients.

There may be other considerations outside the scope of this study that may also impact a surgeon’s decision to resect the entire thymus versus a portion of it. One is in cases of myasthenia gravis, where removal of the entire thymus has been shown to be beneficial for patients’ symptoms and recurrence of disease regardless of the presence of thymoma [20,21]. Therefore, we do not believe that the results of this study should change the treatment course for myasthenic patients. However, patients with thymoma, but without myasthenia gravis or other neoplastic syndromes, may be eligible for a partial thymectomy. Some studies have suggested that partial thymectomy may confer lower operative complications or make it more likely that a surgeon will pursue a minimally invasive approach [8,18,22]. Another instance where partial thymectomy may be considered is a small well-circumscribed 2 cm thymoma on the inferior- and peripheral-most portion of the thymus without any involvement of the pericardium or diaphragm. It is not clear that such tumors really require the resection of the thymic horns superiorly, including all thymic tissue to the contralateral pleural space. There are valid theoretical benefits for either approach, removing the entire thymus to prevent thymoma recurrence or preserve part of the thymus to prevent the reported increase in incidence of extra-thymic malignancy and/or auto-immune disease.

This study has a few limitations. First and foremost, this is an observational study using data from a national database, which collects data from Commission-on-Cancer-accredited centers. Due to the exclusion of non-accredited centers, this introduces potential selection bias and may limit generalizability. The retrospective nature of the study does not allow us to determine direct causation, or to account for all potential confounding factors or errors in hospital reporting. In particular, the NCDB does not collect data on cases of myasthenia gravis, specific location within the thymus or macroscopic morphology, so these individual variables could not be factored into the analysis directly. However, we did factor in the overall comorbidity burden, microscopic tumor morphology, and stage. We believe that the partial and total thymectomy groups being similar in almost all demographic and clinical characteristics, in addition to our propensity score matching and inclusion of only minimally invasive operations, make the results as robust as possible. That said, thymoma is an uncommon disease and a randomized trial is unlikely to happen, making this national database an appropriate data source to answer this question. Second, the NCDB does not provide data on recurrence or disease-free survival. Thymomas are slow-growing tumors with the potential for multifocal, synchronous disease and recurrence. Therefore, further studies may be needed to assess recurrence with both resection extents. However, the absence of an overall survival difference is reassuring. Prior smaller studies have shown no difference in recurrence rates between partial and total thymectomy [23,24]. Additionally, median time to recurrence in prior studies was ~52 months, which falls well within our study period [23]. Therefore, if these recurrences played a clinically significant role in survival, it is likely that we would have seen that effect within our 10-year study period. Third, we do not have information on certain operative factors, such as operative time, estimated blood loss or chest tube duration, so we cannot fully compare the surgical burden between groups. Similarly, the database does not contain granular data on complications and reasons for readmission. Institutional studies where individual patient charts and notes are accessible may be better suited for studying more specific details. Notwithstanding these limitations, we believe our results are important to practicing thoracic surgeons and can help improve shared decision making when discussing operative options with patients.

## 5. Conclusions

In this largest US-hospital-based national study on partial versus total thymectomy, patients with early-stage thymoma undergoing partial thymectomy had comparable peri-operative outcomes and long-term survival to those undergoing total thymectomy. In light of recent attention to the potential importance of the thymus gland, patients without myasthenia gravis who are eligible for minimally invasive resection of early-stage thymomas should be counseled on their options for optimal shared-decision making.

## Figures and Tables

**Table 1:** Baseline patient and tumor characteristics in patients undergoing partial versus total thymectomy in unmatched and matched analyses.

	Unmatched				Matched			
	Partial Thymectomy (N = 495)	Total Thymectomy (N = 1103)	*p*-Value	Standardized Bias|%|	Partial Thymectomy (N = 409)	Total Thymectomy (N = 409)	*p*-Value	Standardized Bias|%|
**Sex**, female	266(53.7%)	589(53.4%)	0.914	2.9%	226(55.3%)	224(54.8%)	0.944	0.1%
**Age**, mean (SD)	63.7(12.2)	61.1(13.3)	<0.001	17.6%	63.4(12.2)	63.4(12.1)	0.970	0.2%
**Race**			0.921	4.6%			0.990	2.6%
White	369(74.5%)	816(74.0%)			306(74.8%)	308(75.3%)		
Black	62(12.5%)	151(13.7%)			52(12.7%)	53(13.0%)		
Asian	48(9.7%)	103(9.3%)			39(9.5%)	36(8.8%)		
Other	16(3.2%)	33(3%)			12(2.9%)	12(2.9%)		
**Ethnicity**, Hispanic	36(7.3%)	83(7.5%)	0.918	4.2%	28(6.9%)	26(6.4%)	0.888	2.0%
**Charlson-Deyo Index**			0.742	6.0%			0.953	2.4%
0	381(77.0%)	833(75.5%)			313(76.5%)	317(77.5%)		
1	77(15.6%)	189(17.1%)			63(15.4%)	60(14.7%)		
2+	37(7.5%)	81(7.3%)			33(8.1%)	32(7.8%)		
**Tumor Size**, mm (SD)	48.7(26.4)	50.4(28.0)	0.455	7.0%	48.4(26.7)	49.6(26.6)	0.512	4.3%
**Histology**			0.587	9.4%			>0.999	2.5%
Type A	71(14.3%)	151(13.7%)			62(15.2%)	59(14.4%)		
Type AB	182(36.8%)	380(34.5%)			148(36.2%)	148(36.2%)		
Type B1	71(14.3%)	185(16.8%)			57(13.9%)	57(13.9%)		
Type B2	80(16.2%)	193(17.5%)			69(16.9%)	71(17.4%)		
Type B3	32(6.5%)	55(5%)			23(5.6%)	24(5.9%)		
Thymoma, NOS	59(11.9%)	139(12.6%)			50(12.2%)	50(12.2%)		
**Primary Payor**			0.063	18.4%			0.986	5.8%
Private Insurance	221(44.6%)	530(48.1%)			190(46.5%)	187(45.7%)		
Medicare	239(48.3%)	458(41.5%)			191(46.7%)	189(46.2%)		
Medicaid/Government	26(5.2%)	89(8.1%)			17(4.2%)	20(4.9%)		
Not Insured	5(1%)	20(1.8%)			3(0.7%)	3(0.7%)		
Unknown	4(0.8%)	6(0.5%)			4(1.0%)	4(1%)		
**Median Income Quartiles**			0.330	11.1%			0.707	3.1%
<$30,000	26(6.1%)	81(8.9%)			25(6.1%)	26(6.4%)		
$30,000–$34,999	59(13.9%)	112(12.3%)			57(13.9%)	54(13.2%)		
$35,000–$45,999	103(24.3%)	219(24.1%)			101(24.7%)	98(24.0%)		
≥$46,000	235(55.6%)	496(54.6%)			226(55.3%)	231(56.5%)		
**Center Type**			0.058	13.1%			0.622	3.9%
Academic	264(53.3%)	531(48.1%)			224(54.8%)	232(56.7%)		
Non-academic	231(46.7%)	572(51.9%)			185(45.2%)	177(43.3%)		
**Approach**			0.135				0.937	
Robotic	333(67.3%)	783(71.0%)			277(67.7%)	280(68.5%)		
VATS	162(32.7%)	320(29.0%)			132(32.3%)	129(31.5%)		

SD—standard deviation, mm—millimeters, NOS—not otherwise specified, VATS—video-assisted thoracoscopic surgery.

**Table 2:** Short-term outcomes in patients undergoing partial versus total thymectomy in unmatched and matched analyses.

	Unmatched Cohort			Matched Cohort		
	Partial Thymectomy	Total Thymectomy	*p* Value	Partial Thymectomy	Total Thymectomy	*p* Value
**30-Day Mortality**	4 (0.8%)	7 (0.6%)	0.747	4 (1.0%)	4 (1.0%)	>0.999
**90-Day Mortality**	4 (0.8%)	9 (0.8%)	>0.999	4 (1.0%)	4 (1.0%)	>0.999
**Resection Margins**			0.386			0.905
Positive	47 (9.9%)	90 (8.5%)		38 (9.7%)	40 (10.1%)	
Negative	428 (90.1%)	963 (91.5%)		356 (90.3%)	356 (89.9%)	
**Length of Stay**, median days (IQR)	2 (1–3)	2 (1–3)	0.943	2 (1–3)	2 (1–3)	0.315
**Readmission within 30 days**	8 (1.6%)	24 (2.2%)	0.564	7 (1.7%)	7 (1.7%)	>0.999

IQR—interquartile range.

**Table 3:** Cox proportional hazards model predicting overall survival.

Variable	aHR	*p*-Value	95% CI
Partial Thymectomy	1.00	0.976	(0.68–1.48)
Negative Margins(R0)	0.91	0.761	(0.54–1.58)
Tumor Size(mm)	1.01	0.004	(1.00–1.01)
Age	1.06	<0.001	(1.04–1.08)
Sex (Female)	0.76	0.146	(0.53–1.10)
Histology			
A	Reference		
AB	0.93	0.773	(0.55–1.57)
B1	0.74	0.392	(0.38–1.46)
B2	0.96	0.899	(0.51–1.82)
B3	1.53	0.243	(0.75–3.12)
NOS	0.91	0.790	(0.46–1.81)
Race			
White	Reference		
Black	1.07	0.810	(0.59–1.96)
Asian	0.91	0.775	(0.47–1.77)
Other	0.98	0.981	(0.24–4.06)
Hispanic	2.20	0.022	(1.12–4.31)
Academic Center	0.66	0.030	(0.46–0.96)
Charlson-Deyo Index			
0	Reference		
1	0.93	0.788	(0.58–1.51)
2+	2.91	<0.001	(1.73–4.88)

aHR—adjusted hazard ratio, CI—confidence interval, R0—negative resection margin, NOS—not otherwise specified.

## Data Availability

Data are available directly from the National Cancer Database to those at CoC-accredited institutions. Data may also be made available from the corresponding author upon reasonable request.

## Supplementary Materials

The following supporting information can be downloaded at: https://www.mdpi.com/article/10.3390/cancers17152518/s1, Table S1: Demographics and short-term outcomes of robotic and thoracoscopic surgery for early-stage thymoma; Figure S1: Love plot comparing standardized bias before and after propensity score matching for all covariates; Figure S2: (**A**) Bihistogram before propensity score matching; (**B**) Bihistogram after propensity score matching.

## Abbreviations

The following abbreviations are used in this manuscript:

WHO	World Health Organization
*NEJM*	*New England Journal of Medicine*
NCDB	National Cancer Database
TNM	Tumor node metastasis
AJCC	American Joint Committee on Cancer
aHR	Adjusted hazard ratio
