# Supplementary material for: Minimally Invasive Total Versus Partial Thymectomy for Early-Stage Thymoma
Source: Cancers (Basel). 2025 Jul 30;17(15):2518. doi: 10.3390/cancers17152518 (PMC12346178; doi:10.3390/cancers17152518)

Supplemental Table S1. Demographics and short-term outcome comparison between robotic and video-assisted thoracoscopic surgery (VATS) for early-stage thymoma

Demographic	Robotic Thymectomy (N= 1,116)	Thoracoscopic Thymectomy (N= 482)	P Value
**Sex**, female	603 (54%)	252 (52.3%)	0.548
**Age**, mean (SD)	62.0 (12.8)	61.9 (13.4)	0.962
**Race**			0.862
White	831 (74.5%)	354 (73.4%)	
Black	150 (13.4%)	63 (13.1%)	
Asian	101 (9.1%)	50 (10.4%)	
Other	34 (3%)	15 (3.1%)	
**Ethnicity**, Hispanic	88 (7.9%)	31 (6.4%)	0.351
**Charlson-Deyo Comorbidity Index**			0.131
0	833 (74.6%)	381 (79%)	
1	193 (17.3%)	73 (15.1%)	
2+	90 (8.1%)	28 (5.8%)	
**Tumor Size**, mm (SD)	48.9 (26.0)	53.0 (30.9)	0.209
**Histology**			0.204
Type A	165 (14.8%)	57 (11.8%)	
Type AB	385 (34.5%)	177 (36.7%)	
Type B1	173 (15.5%)	83 (17.2%)	
Type B2	193 (17.3%)	80 (16.6%)	
Type B3	54 (4.8%)	33 (6.8%)	
Type NOS	146 (13.1%)	52 (10.8%)	
**Primary Payor**			0.032
Private Insurance	540 (48.4%)	211 (43.8%)	
Medicare	488 (43.7%)	209 (43.4%)	
Medicaid/Other Government	65 (5.8%)	50 (10.4%)	
Not Insured	16 (1.4%)	9 (1.9%)	
Insurance Status Unknown	7 (0.6%)	3 (0.6%)	
**Median Income Quartiles**			0.204
< $30,000	82 (8.9%)	25 (6.1%)	
$30,000-$34,999	114 (12.4%)	57 (13.9%)	
$35,000-$45,999	229 (24.9%)	93 (22.6%)	
>= $46,000	495 (53.8%)	236 (57.4%)	
**Center Type**			0.114
Academic Center	570 (51.1%)	225 (46.7%)	
All other centers	546 (48.9%)	257 (53.3%)	

Short-Term Outcomes			
**30 Day Mortality**	9 (0.8%)	2 (0.4%)	0.521
**90 Day Mortality**	11 (1%)	2 (0.4%)	0.366
**Resection Margins**			0.125
Positive	88 (8.2%)	49 (10.7%)	
Negative	981 (91.8%)	410 (89.3%)	
**Length of Stay**, median days (IQR)	2 (1-3)	2 (1-3)	0.978
**Readmission within 30 days**	17 (1.5%)	15 (3.1%)	0.034

SD – standard deviation, mm – millimeter, IQR – interquartile range

Supplemental Figure S1. Love plot comparing standardized mean difference before and after propensity score matching for all covariates.

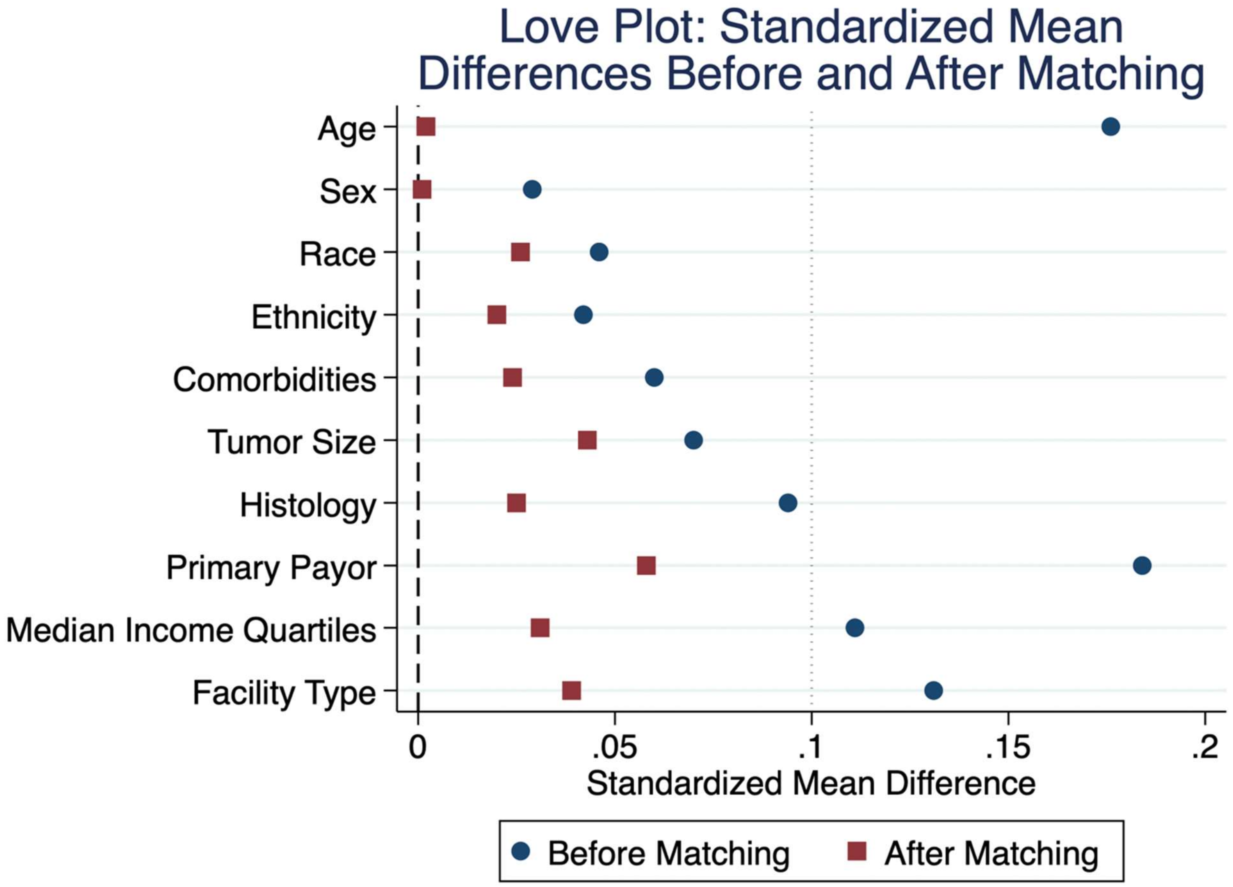

Supplemental Figure S2. **(A)** Bihistogram before propensity score matching; **(B)** Bihistogram after propensity score matching

A

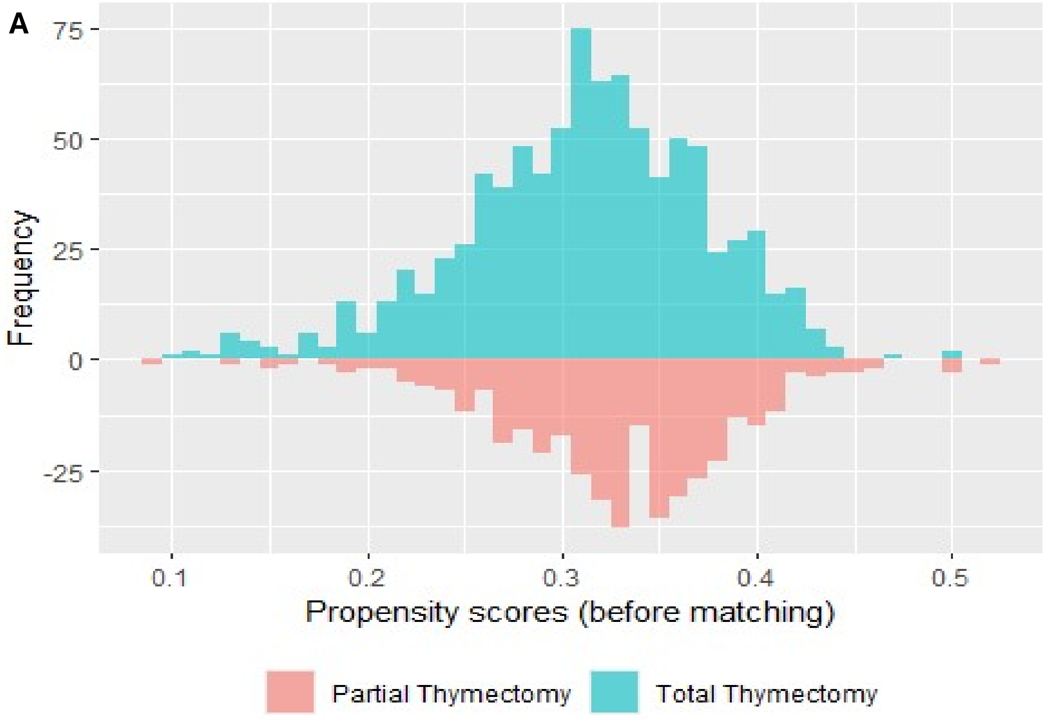

B

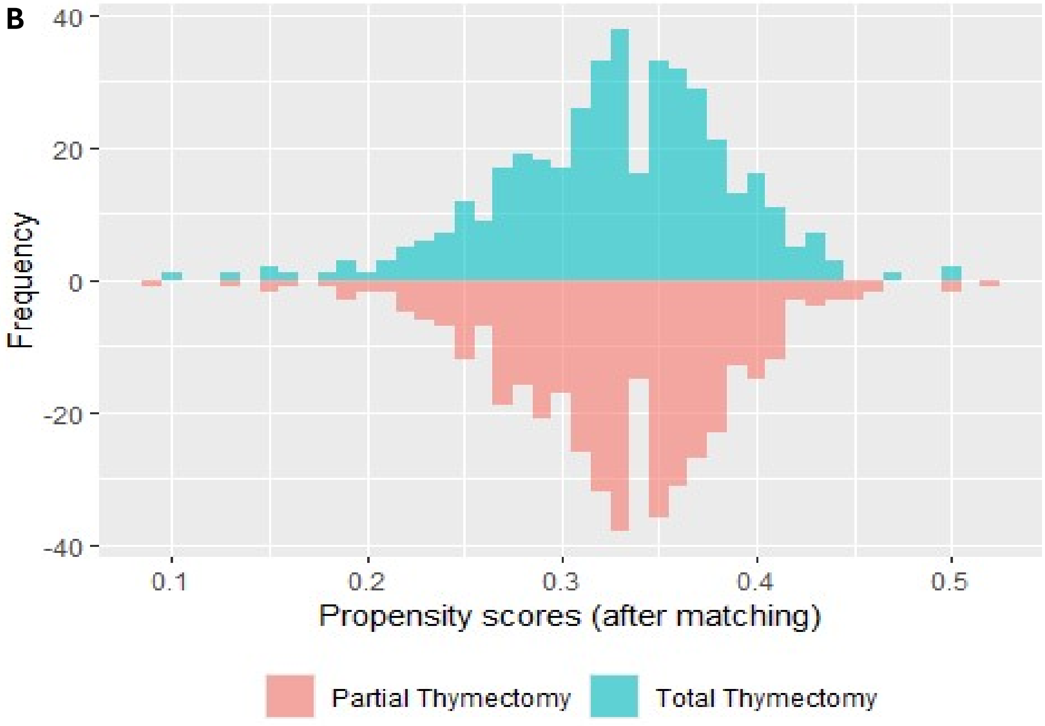

Supplement: Supplementary file 1 [file cancers-17-02518-s001.zip › cancers-3723049-supplementary.pdf]
